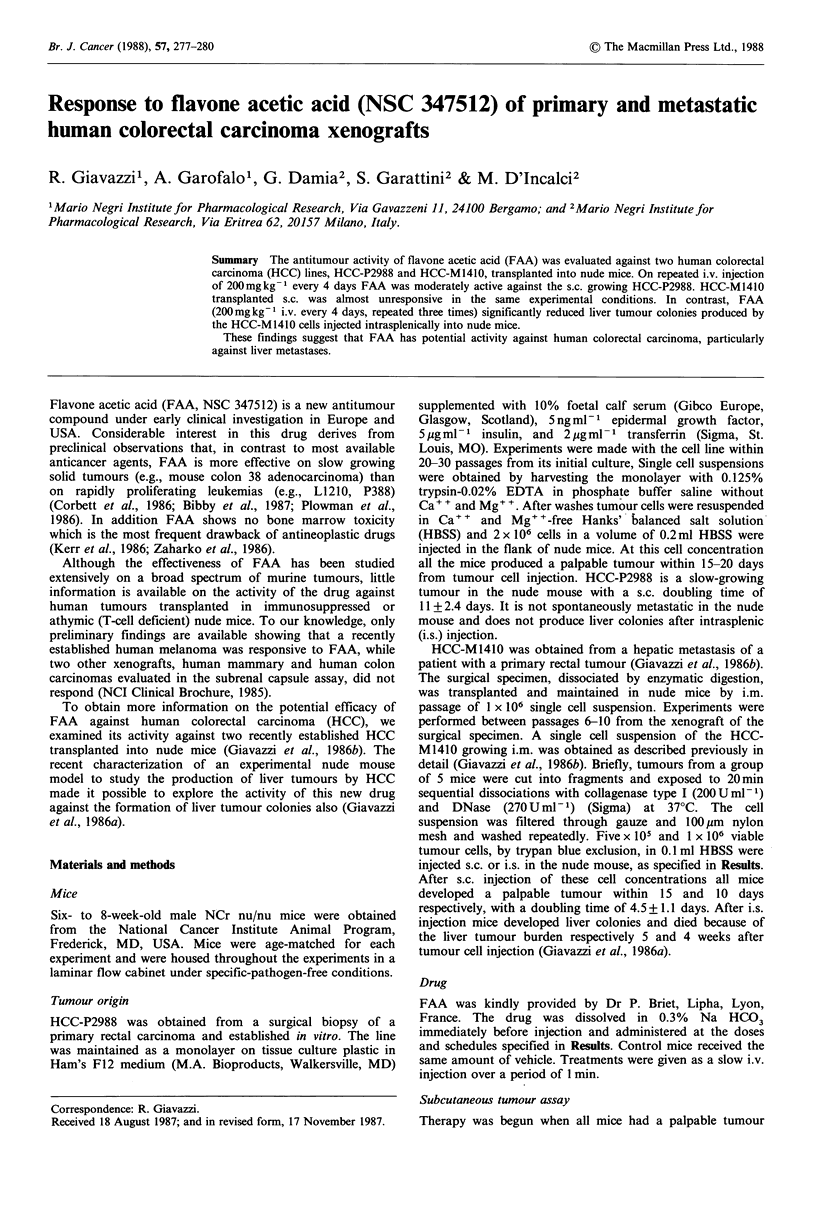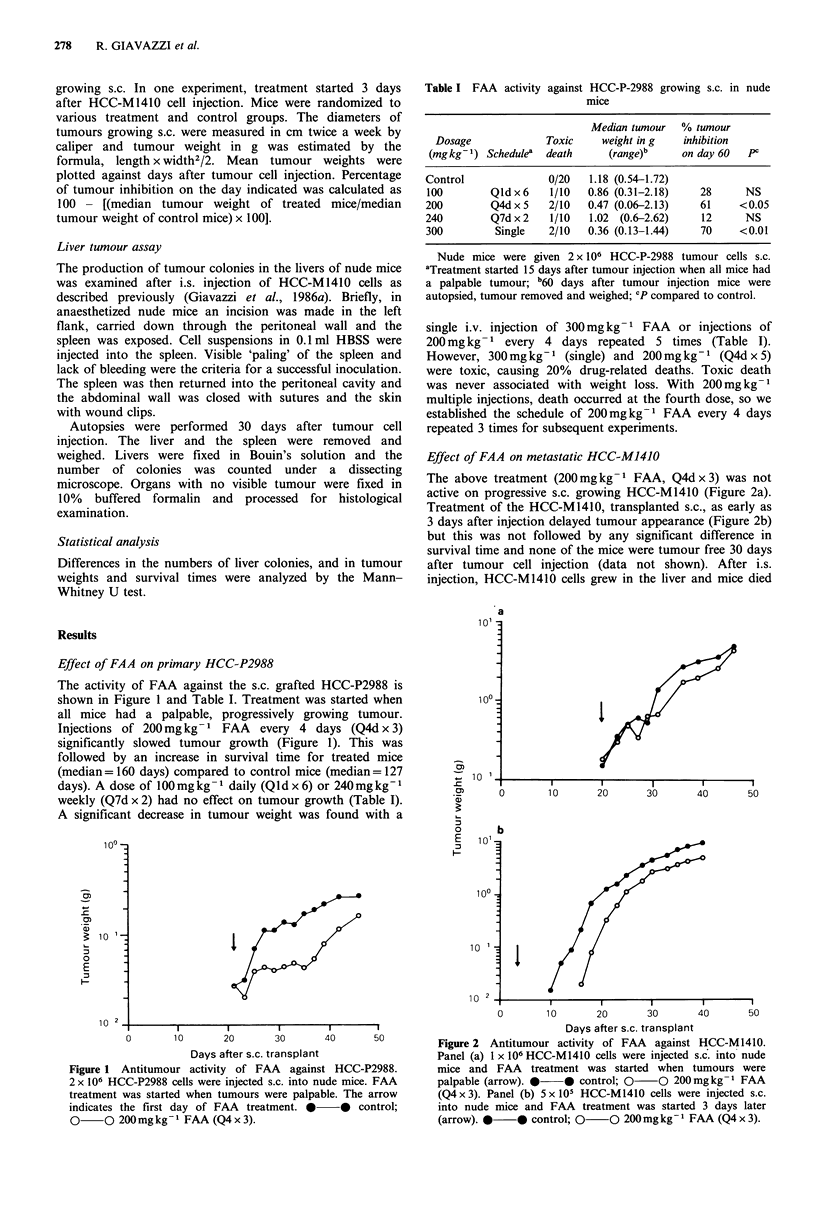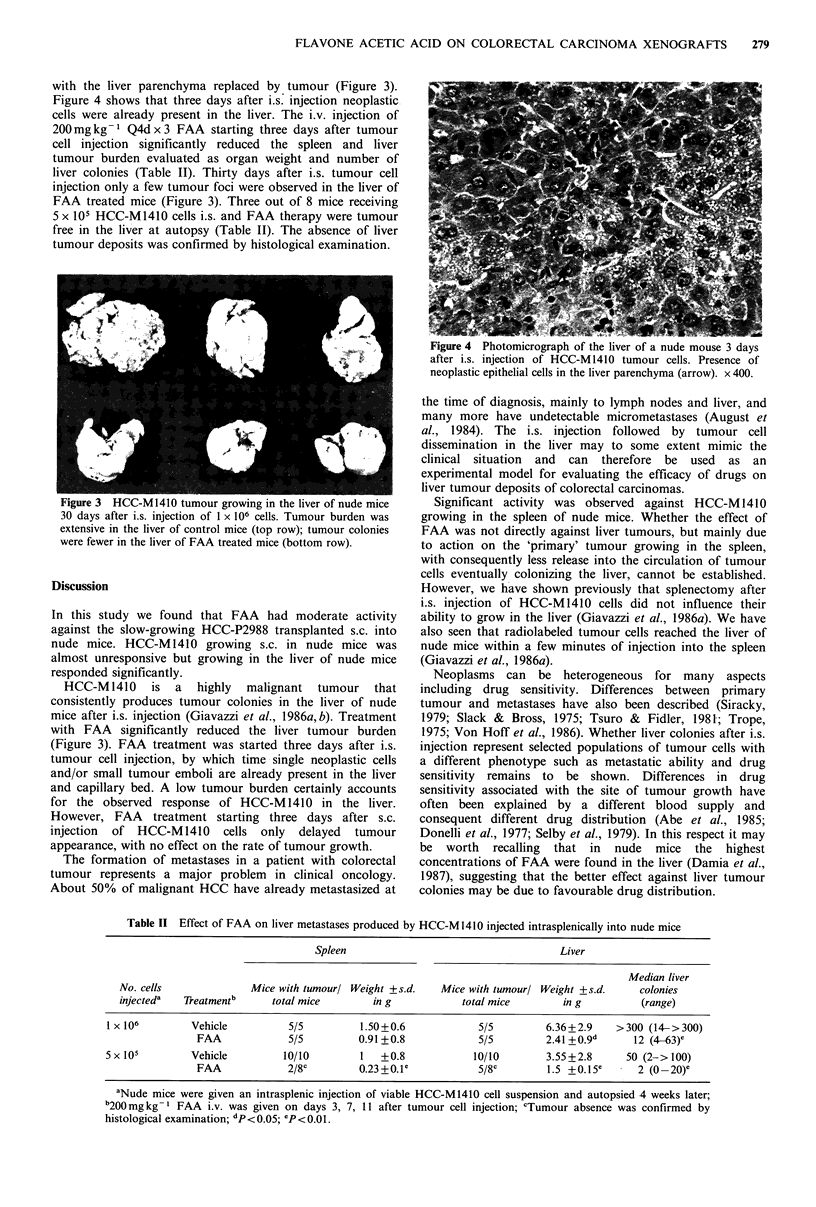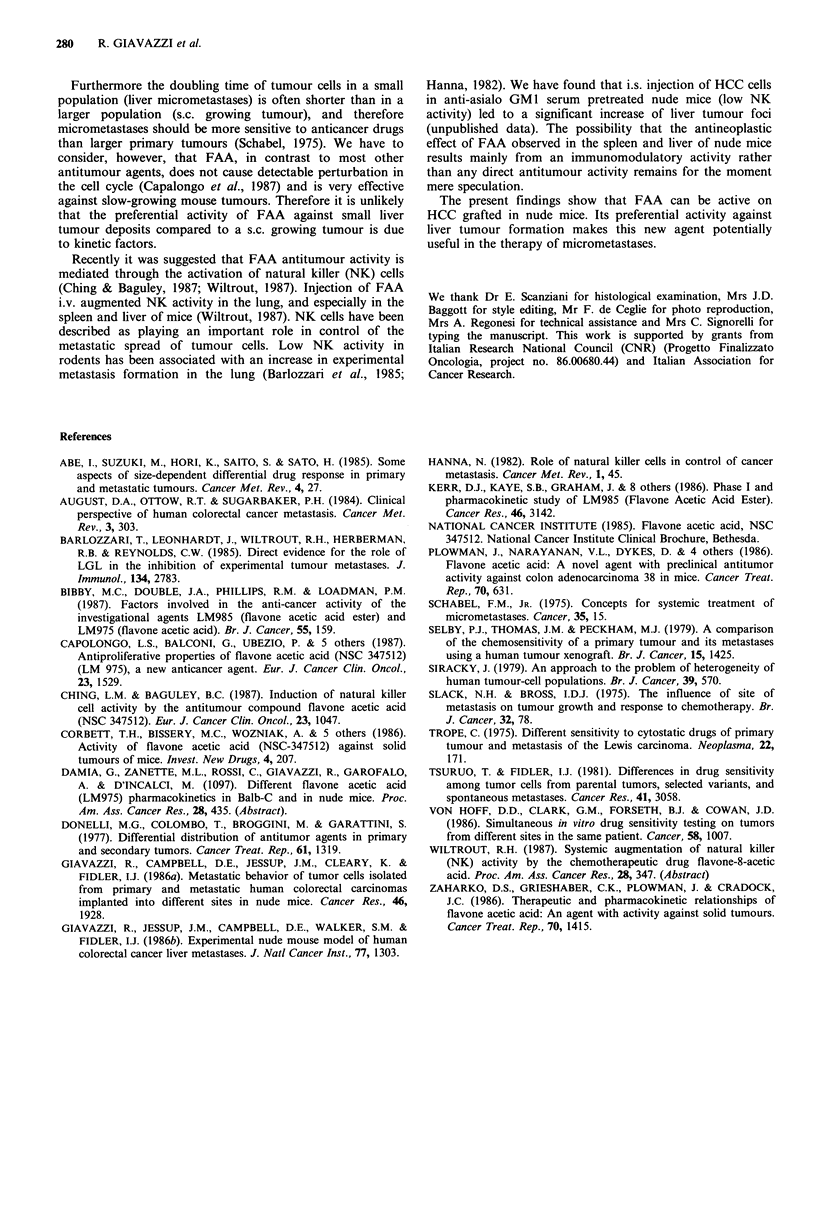# Response to flavone acetic acid (NSC 347512) of primary and metastatic human colorectal carcinoma xenografts.

**DOI:** 10.1038/bjc.1988.59

**Published:** 1988-03

**Authors:** R. Giavazzi, A. Garofalo, G. Damia, S. Garattini, M. D'Incalci

**Affiliations:** Mario Negri Institute for Pharmacological Research, Bergamo, Italy.

## Abstract

**Images:**


*Br. J. Cancer* (1988), **57**, 277–280 © The Macmillan Press Ltd., 1988

### Response to flavone acetic acid (NSC 347512) of primary and metastatic human colorectal carcinoma xenografts

R. Giavazzi[1], A. Garofalo[1], G. Damia[2], S. Garattini[2] & M. D'Incalci[2]

[1]*Mario Negri Institute for Pharmacological Research, Via Gavazzeni 11, 24100 Bergamo; and* [2]*Mario Negri Institute for Pharmacological Research, Via Eritrea 62, 20157 Milano, Italy.*

**Summary** The antitumour activity of flavone acetic acid (FAA) was evaluated against two human colorectal carcinoma (HCC) lines, HCC-P2988 and HCC-M1410, transplanted into nude mice. On repeated i.v. injection of 200 mg kg$^{-1}$ every 4 days FAA was moderately active against the s.c. growing HCC-P2988. HCC-M1410 transplanted s.c. was almost unresponsive in the same experimental conditions. In contrast, FAA (200 mg kg$^{-1}$ i.v. every 4 days, repeated three times) significantly reduced liver tumour colonies produced by the HCC-M1410 cells injected intrasplenically into nude mice.

These findings suggest that FAA has potential activity against human colorectal carcinoma, particularly against liver metastases.

Flavone acetic acid (FAA, NSC 347512) is a new antitumour compound under early clinical investigation in Europe and USA. Considerable interest in this drug derives from preclinical observations that, in contrast to most available anticancer agents, FAA is more effective on slow growing solid tumours (e.g., mouse colon 38 adenocarcinoma) than on rapidly proliferating leukemias (e.g., L1210, P388) (Corbett *et al.*, 1986; Bibby *et al.*, 1987; Plowman *et al.*, 1986). In addition FAA shows no bone marrow toxicity which is the most frequent drawback of antineoplastic drugs (Kerr *et al.*, 1986; Zaharko *et al.*, 1986).

Although the effectiveness of FAA has been studied extensively on a broad spectrum of murine tumours, little information is available on the activity of the drug against human tumours transplanted in immunosuppressed or athymic (T-cell deficient) nude mice. To our knowledge, only preliminary findings are available showing that a recently established human melanoma was responsive to FAA, while two other xenografts, human mammary and human colon carcinomas evaluated in the subrenal capsule assay, did not respond (NCI Clinical Brochure, 1985).

To obtain more information on the potential efficacy of FAA against human colorectal carcinoma (HCC), we examined its activity against two recently established HCC transplanted into nude mice (Giavazzi *et al.*, 1986*b*). The recent characterization of an experimental nude mouse model to study the production of liver tumours by HCC made it possible to explore the activity of this new drug against the formation of liver tumour colonies also (Giavazzi *et al.*, 1986*a*).

### Materials and methods

### *Mice*

Six- to 8-week-old male NCr nu/nu mice were obtained from the National Cancer Institute Animal Program, Frederick, MD, USA. Mice were age-matched for each experiment and were housed throughout the experiments in a laminar flow cabinet under specific-pathogen-free conditions.

### *Tumour origin*

HCC-P2988 was obtained from a surgical biopsy of a primary rectal carcinoma and established *in vitro*. The line was maintained as a monolayer on tissue culture plastic in Ham's F12 medium (M.A. Bioproducts, Walkersville, MD) supplemented with 10% foetal calf serum (Gibco Europe, Glasgow, Scotland), 5 ng ml$^{-1}$ epidermal growth factor, 5 $\mu$g ml$^{-1}$ insulin, and 2 $\mu$g ml$^{-1}$ transferrin (Sigma, St. Louis, MO). Experiments were made with the cell line within 20–30 passages from its initial culture, Single cell suspensions were obtained by harvesting the monolayer with 0.125% trypsin-0.02% EDTA in phosphate buffer saline without $Ca^{++}$ and $Mg^{++}$. After washes tumour cells were resuspended in $Ca^{++}$ and $Mg^{++}$-free Hanks' balanced salt solution (HBSS) and $2\times10^6$ cells in a volume of 0.2 ml HBSS were injected in the flank of nude mice. At this cell concentration all the mice produced a palpable tumour within 15–20 days from tumour cell injection. HCC-P2988 is a slow-growing tumour in the nude mouse with a s.c. doubling time of $11\pm2.4$ days. It is not spontaneously metastatic in the nude mouse and does not produce liver colonies after intrasplenic (i.s.) injection.

HCC-M1410 was obtained from a hepatic metastasis of a patient with a primary rectal tumour (Giavazzi *et al.*, 1986*b*). The surgical specimen, dissociated by enzymatic digestion, was transplanted and maintained in nude mice by i.m. passage of $1\times10^6$ single cell suspension. Experiments were performed between passages 6–10 from the xenograft of the surgical specimen. A single cell suspension of the HCC-M1410 growing i.m. was obtained as described previously in detail (Giavazzi *et al.*, 1986*b*). Briefly, tumours from a group of 5 mice were cut into fragments and exposed to 20 min sequential dissociations with collagenase type I (200 U ml$^{-1}$) and DNase (270 U ml$^{-1}$) (Sigma) at 37°C. The cell suspension was filtered through gauze and 100 $\mu$m nylon mesh and washed repeatedly. Five $\times10^5$ and $1\times10^6$ viable tumour cells, by trypan blue exclusion, in 0.1 ml HBSS were injected s.c. or i.s. in the nude mouse, as specified in **Results**. After s.c. injection of these cell concentrations all mice developed a palpable tumour within 15 and 10 days respectively, with a doubling time of $4.5\pm1.1$ days. After i.s. injection mice developed liver colonies and died because of the liver tumour burden respectively 5 and 4 weeks after tumour cell injection (Giavazzi *et al.*, 1986*a*).

### *Drug*

FAA was kindly provided by Dr P. Briet, Lipha, Lyon, France. The drug was dissolved in 0.3% Na $HCO_3$ immediately before injection and administered at the doses and schedules specified in **Results**. Control mice received the same amount of vehicle. Treatments were given as a slow i.v. injection over a period of 1 min.

### *Subcutaneous tumour assay*

Therapy was begun when all mice had a palpable tumour

Correspondence: R. Giavazzi.
Received 18 August 1987; and in revised form, 17 November 1987.

growing s.c. In one experiment, treatment started 3 days after HCC-M1410 cell injection. Mice were randomized to various treatment and control groups. The diameters of tumours growing s.c. were measured in cm twice a week by caliper and tumour weight in g was estimated by the formula, length × width$^2$/2. Mean tumour weights were plotted against days after tumour cell injection. Percentage of tumour inhibition on the day indicated was calculated as 100 − [(median tumour weight of treated mice/median tumour weight of control mice) × 100].

*Liver tumour assay*

The production of tumour colonies in the livers of nude mice was examined after i.s. injection of HCC-M1410 cells as described previously (Giavazzi *et al.*, 1986*a*). Briefly, in anaesthetized nude mice an incision was made in the left flank, carried down through the peritoneal wall and the spleen was exposed. Cell suspensions in 0.1 ml HBSS were injected into the spleen. Visible 'paling' of the spleen and lack of bleeding were the criteria for a successful inoculation. The spleen was then returned into the peritoneal cavity and the abdominal wall was closed with sutures and the skin with wound clips.

Autopsies were performed 30 days after tumour cell injection. The liver and the spleen were removed and weighed. Livers were fixed in Bouin's solution and the number of colonies was counted under a dissecting microscope. Organs with no visible tumour were fixed in 10% buffered formalin and processed for histological examination.

*Statistical analysis*

Differences in the numbers of liver colonies, and in tumour weights and survival times were analyzed by the Mann–Whitney U test.

### Results

*Effect of FAA on primary HCC-P2988*

The activity of FAA against the s.c. grafted HCC-P2988 is shown in Figure 1 and Table I. Treatment was started when all mice had a palpable, progressively growing tumour. Injections of 200 mg kg$^{-1}$ FAA every 4 days (Q4d × 3) significantly slowed tumour growth (Figure 1). This was followed by an increase in survival time for treated mice (median = 160 days) compared to control mice (median = 127 days). A dose of 100 mg kg$^{-1}$ daily (Q1d × 6) or 240 mg kg$^{-1}$ weekly (Q7d × 2) had no effect on tumour growth (Table I). A significant decrease in tumour weight was found with a

**Figure 1** Antitumour activity of FAA against HCC-P2988. 2 × 10$^6$ HCC-P2988 cells were injected s.c. into nude mice. FAA treatment was started when tumours were palpable. The arrow indicates the first day of FAA treatment. ●——● control; ○——○ 200 mg kg$^{-1}$ FAA (Q4 × 3).

**Table I** FAA activity against HCC-P-2988 growing s.c. in nude mice

| Dosage (mg kg$^{-1}$) | Schedule[a] | Toxic death | Median tumour weight in g (range)[b] | % tumour inhibition on day 60 | P[c] |
|---|---|---|---|---|---|
| Control | | 0/20 | 1.18 (0.54–1.72) | | |
| 100 | Q1d × 6 | 1/10 | 0.86 (0.31–2.18) | 28 | NS |
| 200 | Q4d × 5 | 2/10 | 0.47 (0.06–2.13) | 61 | <0.05 |
| 240 | Q7d × 2 | 1/10 | 1.02 (0.6–2.62) | 12 | NS |
| 300 | Single | 2/10 | 0.36 (0.13–1.44) | 70 | <0.01 |

Nude mice were given 2 × 10$^6$ HCC-P-2988 tumour cells s.c. [a]Treatment started 15 days after tumour injection when all mice had a palpable tumour; [b]60 days after tumour injection mice were autopsied, tumour removed and weighed; [c]*P* compared to control.

single i.v. injection of 300 mg kg$^{-1}$ FAA or injections of 200 mg kg$^{-1}$ every 4 days repeated 5 times (Table I). However, 300 mg kg$^{-1}$ (single) and 200 mg kg$^{-1}$ (Q4d × 5) were toxic, causing 20% drug-related deaths. Toxic death was never associated with weight loss. With 200 mg kg$^{-1}$ multiple injections, death occurred at the fourth dose, so we established the schedule of 200 mg kg$^{-1}$ FAA every 4 days repeated 3 times for subsequent experiments.

*Effect of FAA on metastatic HCC-M1410*

The above treatment (200 mg kg$^{-1}$ FAA, Q4d × 3) was not active on progressive s.c. growing HCC-M1410 (Figure 2a). Treatment of the HCC-M1410, transplanted s.c., as early as 3 days after injection delayed tumour appearance (Figure 2b) but this was not followed by any significant difference in survival time and none of the mice were tumour free 30 days after tumour cell injection (data not shown). After i.s. injection, HCC-M1410 cells grew in the liver and mice died

**Figure 2** Antitumour activity of FAA against HCC-M1410. Panel (a) 1 × 10$^6$ HCC-M1410 cells were injected s.c. into nude mice and FAA treatment was started when tumours were palpable (arrow). ●——● control; ○——○ 200 mg kg$^{-1}$ FAA (Q4 × 3). Panel (b) 5 × 10$^5$ HCC-M1410 cells were injected s.c. into nude mice and FAA treatment was started 3 days later (arrow). ●——● control; ○——○ 200 mg kg$^{-1}$ FAA (Q4 × 3).

with the liver parenchyma replaced by tumour (Figure 3). Figure 4 shows that three days after i.s. injection neoplastic cells were already present in the liver. The i.v. injection of 200 mg kg$^{-1}$ Q4d × 3 FAA starting three days after tumour cell injection significantly reduced the spleen and liver tumour burden evaluated as organ weight and number of liver colonies (Table II). Thirty days after i.s. tumour cell injection only a few tumour foci were observed in the liver of FAA treated mice (Figure 3). Three out of 8 mice receiving $5 \times 10^5$ HCC-M1410 cells i.s. and FAA therapy were tumour free in the liver at autopsy (Table II). The absence of liver tumour deposits was confirmed by histological examination.

**Figure 3** HCC-M1410 tumour growing in the liver of nude mice 30 days after i.s. injection of $1 \times 10^6$ cells. Tumour burden was extensive in the liver of control mice (top row); tumour colonies were fewer in the liver of FAA treated mice (bottom row).

**Figure 4** Photomicrograph of the liver of a nude mouse 3 days after i.s. injection of HCC-M1410 tumour cells. Presence of neoplastic epithelial cells in the liver parenchyma (arrow). × 400.

### Discussion

In this study we found that FAA had moderate activity against the slow-growing HCC-P2988 transplanted s.c. into nude mice. HCC-M1410 growing s.c. in nude mice was almost unresponsive but growing in the liver of nude mice responded significantly.

HCC-M1410 is a highly malignant tumour that consistently produces tumour colonies in the liver of nude mice after i.s. injection (Giavazzi *et al.*, 1986*a*, *b*). Treatment with FAA significantly reduced the liver tumour burden (Figure 3). FAA treatment was started three days after i.s. tumour cell injection, by which time single neoplastic cells and/or small tumour emboli are already present in the liver and capillary bed. A low tumour burden certainly accounts for the observed response of HCC-M1410 in the liver. However, FAA treatment starting three days after s.c. injection of HCC-M1410 cells only delayed tumour appearance, with no effect on the rate of tumour growth.

The formation of metastases in a patient with colorectal tumour represents a major problem in clinical oncology. About 50% of malignant HCC have already metastasized at the time of diagnosis, mainly to lymph nodes and liver, and many more have undetectable micrometastases (August *et al.*, 1984). The i.s. injection followed by tumour cell dissemination in the liver may to some extent mimic the clinical situation and can therefore be used as an experimental model for evaluating the efficacy of drugs on liver tumour deposits of colorectal carcinomas.

Significant activity was observed against HCC-M1410 growing in the spleen of nude mice. Whether the effect of FAA was not directly against liver tumours, but mainly due to action on the 'primary' tumour growing in the spleen, with consequently less release into the circulation of tumour cells eventually colonizing the liver, cannot be established. However, we have shown previously that splenectomy after i.s. injection of HCC-M1410 cells did not influence their ability to grow in the liver (Giavazzi *et al.*, 1986*a*). We have also seen that radiolabeled tumour cells reached the liver of nude mice within a few minutes of injection into the spleen (Giavazzi *et al.*, 1986*a*).

Neoplasms can be heterogeneous for many aspects including drug sensitivity. Differences between primary tumour and metastases have also been described (Siracky, 1979; Slack & Bross, 1975; Tsuro & Fidler, 1981; Trope, 1975; Von Hoff *et al.*, 1986). Whether liver colonies after i.s. injection represent selected populations of tumour cells with a different phenotype such as metastatic ability and drug sensitivity remains to be shown. Differences in drug sensitivity associated with the site of tumour growth have often been explained by a different blood supply and consequent different drug distribution (Abe *et al.*, 1985; Donelli *et al.*, 1977; Selby *et al.*, 1979). In this respect it may be worth recalling that in nude mice the highest concentrations of FAA were found in the liver (Damia *et al.*, 1987), suggesting that the better effect against liver tumour colonies may be due to favourable drug distribution.

**Table II** Effect of FAA on liver metastases produced by HCC-M1410 injected intrasplenically into nude mice

| | | *Spleen* | | *Liver* | | |
|---|---|---|---|---|---|---|
| *No. cells injected*[a] | *Treatment*[b] | *Mice with tumour/ total mice* | *Weight ± s.d. in g* | *Mice with tumour/ total mice* | *Weight ± s.d. in g* | *Median liver colonies (range)* |
| $1 \times 10^6$ | Vehicle | 5/5 | 1.50 ± 0.6 | 5/5 | 6.36 ± 2.9 | > 300 (14–> 300) |
| | FAA | 5/5 | 0.91 ± 0.8 | 5/5 | 2.41 ± 0.9[d] | 12 (4–63)[e] |
| $5 \times 10^5$ | Vehicle | 10/10 | 1 ± 0.8 | 10/10 | 3.55 ± 2.8 | 50 (2–> 100) |
| | FAA | 2/8[c] | 0.23 ± 0.1[e] | 5/8[c] | 1.5 ± 0.15[e] | 2 (0–20)[e] |

[a]Nude mice were given an intrasplenic injection of viable HCC-M1410 cell suspension and autopsied 4 weeks later; [b]200 mg kg$^{-1}$ FAA i.v. was given on days 3, 7, 11 after tumour cell injection; [c]Tumour absence was confirmed by histological examination; [d]$P < 0.05$; [e]$P < 0.01$.

Furthermore the doubling time of tumour cells in a small population (liver micrometastases) is often shorter than in a larger population (s.c. growing tumour), and therefore micrometastases should be more sensitive to anticancer drugs than larger primary tumours (Schabel, 1975). We have to consider, however, that FAA, in contrast to most other antitumour agents, does not cause detectable perturbation in the cell cycle (Capalongo *et al.*, 1987) and is very effective against slow-growing mouse tumours. Therefore it is unlikely that the preferential activity of FAA against small liver tumour deposits compared to a s.c. growing tumour is due to kinetic factors.

Recently it was suggested that FAA antitumour activity is mediated through the activation of natural killer (NK) cells (Ching & Baguley, 1987; Wiltrout, 1987). Injection of FAA i.v. augmented NK activity in the lung, and especially in the spleen and liver of mice (Wiltrout, 1987). NK cells have been described as playing an important role in control of the metastatic spread of tumour cells. Low NK activity in rodents has been associated with an increase in experimental metastasis formation in the lung (Barlozzari *et al.*, 1985; Hanna, 1982). We have found that i.s. injection of HCC cells in anti-asialo GM1 serum pretreated nude mice (low NK activity) led to a significant increase of liver tumour foci (unpublished data). The possibility that the antineoplastic effect of FAA observed in the spleen and liver of nude mice results mainly from an immunomodulatory activity rather than any direct antitumour activity remains for the moment mere speculation.

The present findings show that FAA can be active on HCC grafted in nude mice. Its preferential activity against liver tumour formation makes this new agent potentially useful in the therapy of micrometastases.

We thank Dr E. Scanziani for histological examination, Mrs J.D. Baggott for style editing, Mr F. de Ceglie for photo reproduction, Mrs A. Regonesi for technical assistance and Mrs C. Signorelli for typing the manuscript. This work is supported by grants from Italian Research National Council (CNR) (Progetto Finalizzato Oncologia, project no. 86.00680.44) and Italian Association for Cancer Research.

### References

ABE, I., SUZUKI, M., HORI, K., SAITO, S. & SATO, H. (1985). Some aspects of size-dependent differential drug response in primary and metastatic tumours. *Cancer Met. Rev.*, **4**, 27.

AUGUST, D.A., OTTOW, R.T. & SUGARBAKER, P.H. (1984). Clinical perspective of human colorectal cancer metastasis. *Cancer Met. Rev.*, **3**, 303.

BARLOZZARI, T., LEONHARDT, J., WILTROUT, R.H., HERBERMAN, R.B. & REYNOLDS, C.W. (1985). Direct evidence for the role of LGL in the inhibition of experimental tumour metastases. *J. Immunol.*, **134**, 2783.

BIBBY, M.C., DOUBLE, J.A., PHILLIPS, R.M. & LOADMAN, P.M. (1987). Factors involved in the anti-cancer activity of the investigational agents LM985 (flavone acetic acid ester) and LM975 (flavone acetic acid). *Br. J. Cancer*, **55**, 159.

CAPOLONGO, L.S., BALCONI, G., UBEZIO, P. & 5 others (1987). Antiproliferative properties of flavone acetic acid (NSC 347512) (LM 975), a new anticancer agent. *Eur. J. Cancer Clin. Oncol.*, **23**, 1529.

CHING, L.M. & BAGULEY, B.C. (1987). Induction of natural killer cell activity by the antitumour compound flavone acetic acid (NSC 347512). *Eur. J. Cancer Clin. Oncol.*, **23**, 1047.

CORBETT, T.H., BISSERY, M.C., WOZNIAK, A. & 5 others (1986). Activity of flavone acetic acid (NSC-347512) against solid tumours of mice. *Invest. New Drugs*, **4**, 207.

DAMIA, G., ZANETTE, M.L., ROSSI, C., GIAVAZZI, R., GAROFALO, A. & D'INCALCI, M. (1097). Different flavone acetic acid (LM975) pharmacokinetics in Balb-C and in nude mice. *Proc. Am. Ass. Cancer Res.*, **28**, 435. (*Abstract*).

DONELLI, M.G., COLOMBO, T., BROGGINI, M. & GARATTINI, S. (1977). Differential distribution of antitumor agents in primary and secondary tumors. *Cancer Treat. Rep.*, **61**, 1319.

GIAVAZZI, R., CAMPBELL, D.E., JESSUP, J.M., CLEARY, K. & FIDLER, I.J. (1986*a*). Metastatic behavior of tumor cells isolated from primary and metastatic human colorectal carcinomas implanted into different sites in nude mice. *Cancer Res.*, **46**, 1928.

GIAVAZZI, R., JESSUP, J.M., CAMPBELL, D.E., WALKER, S.M. & FIDLER, I.J. (1986*b*). Experimental nude mouse model of human colorectal cancer liver metastases. *J. Natl Cancer Inst.*, **77**, 1303.

HANNA, N. (1982). Role of natural killer cells in control of cancer metastasis. *Cancer Met. Rev.*, **1**, 45.

KERR, D.J., KAYE, S.B., GRAHAM, J. & 8 others (1986). Phase I and pharmacokinetic study of LM985 (Flavone Acetic Acid Ester). *Cancer Res.*, **46**, 3142.

NATIONAL CANCER INSTITUTE (1985). Flavone acetic acid, NSC 347512. National Cancer Institute Clinical Brochure, Bethesda.

PLOWMAN, J., NARAYANAN, V.L., DYKES, D. & 4 others (1986). Flavone acetic acid: A novel agent with preclinical antitumor activity against colon adenocarcinoma 38 in mice. *Cancer Treat. Rep.*, **70**, 631.

SCHABEL, F.M., JR. (1975). Concepts for systemic treatment of micrometastases. *Cancer*, **35**, 15.

SELBY, P.J., THOMAS, J.M. & PECKHAM, M.J. (1979). A comparison of the chemosensitivity of a primary tumour and its metastases using a human tumour xenograft. *Br. J. Cancer*, **15**, 1425.

SIRACKY, J. (1979). An approach to the problem of heterogeneity of human tumour-cell populations. *Br. J. Cancer*, **39**, 570.

SLACK, N.H. & BROSS, I.D.J. (1975). The influence of site of metastasis on tumour growth and response to chemotherapy. *Br. J. Cancer*, **32**, 78.

TROPE, C. (1975). Different sensitivity to cytostatic drugs of primary tumour and metastasis of the Lewis carcinoma. *Neoplasma*, **22**, 171.

TSURUO, T. & FIDLER, I.J. (1981). Differences in drug sensitivity among tumor cells from parental tumors, selected variants, and spontaneous metastases. *Cancer Res.*, **41**, 3058.

VON HOFF, D.D., CLARK, G.M., FORSETH, B.J. & COWAN, J.D. (1986). Simultaneous *in vitro* drug sensitivity testing on tumors from different sites in the same patient. *Cancer*, **58**, 1007.

WILTROUT, R.H. (1987). Systemic augmentation of natural killer (NK) activity by the chemotherapeutic drug flavone-8-acetic acid. *Proc. Am. Ass. Cancer Res.*, **28**, 347. (*Abstract*)

ZAHARKO, D.S., GRIESHABER, C.K., PLOWMAN, J. & CRADOCK, J.C. (1986). Therapeutic and pharmacokinetic relationships of flavone acetic acid: An agent with activity against solid tumours. *Cancer Treat. Rep.*, **70**, 1415.